# Breaking bandwidth limits in transformation optics with Brewster-enhanced metamaterials

**DOI:** 10.1093/nsr/nwag023

**Published:** 2026-01-14

**Authors:** Xiaojun Hu, Yu Luo, Jingxin Tang, Chun Wang, Yuan Gao, Jingjing Zhang, Yi Zhang, Dexin Ye

**Affiliations:** Laboratory of Applied Research on Electromagnetics (ARE), College of Information Science and Electronic Engineering, Zhejiang University, Hangzhou 310027, China; National Key Laboratory of Microwave Photonics, College of Electronic and Information Engineering, Nanjing University of Aeronautics and Astronautics, Nanjing 211106, China; Laboratory of Applied Research on Electromagnetics (ARE), College of Information Science and Electronic Engineering, Zhejiang University, Hangzhou 310027, China; Laboratory of Applied Research on Electromagnetics (ARE), College of Information Science and Electronic Engineering, Zhejiang University, Hangzhou 310027, China; Laboratory of Applied Research on Electromagnetics (ARE), College of Information Science and Electronic Engineering, Zhejiang University, Hangzhou 310027, China; School of Electrical and Electronic Engineering, Shandong University of Technology, Zibo 255000, China; State Key Laboratory of Millimeter Waves, School of Information Science and Engineering, Southeast University, Nanjing 210096, China; Laboratory of Applied Research on Electromagnetics (ARE), College of Information Science and Electronic Engineering, Zhejiang University, Hangzhou 310027, China; Laboratory of Applied Research on Electromagnetics (ARE), College of Information Science and Electronic Engineering, Zhejiang University, Hangzhou 310027, China

**Keywords:** transformation optics, broadband omnidirectional invisibility, full parameter, Brewster effect

## Abstract

Transformation optics (TO) enables unprecedented electromagnetic wave manipulation through the theory of coordinate transformations, yet its practical implementation has been fundamentally constrained by narrowband operation stemming from extreme material requirements. To break this spectral bottleneck, a dual-mode metamaterial architecture synergizing Brewster-angle broadband transmission with Fabry–Pérot multiband resonance is proposed. This approach leverages cascaded impedance-engineered slot cavities—constructed from conventional dielectrics and standard metallic patterning—to achieve transformation invariance while eliminating exotic material needs. This framework enables two spectral functionalities simultaneously in a single platform: (i) omnidirectional multiband operation through discrete resonances and (ii) broadband unidirectional performance via angular-selective Brewster transmission. Experimental validation demonstrates a full-parameter invisibility cloak maintaining >88.4% transmittance across X-band frequencies (7.5–12.5 GHz) with ±70$^\circ$ angular tolerance, alongside a retroreflector achieving near-unity efficiency in X/K bands (12–24 GHz) within ±60$^\circ$ illumination. By resolving the fundamental conflict between bandwidth and geometric complexity in TO designs, this work establishes a scalable paradigm for multifunctional wave-control devices spanning radar stealth to next-generation communications.

## Teaser

This study overcomes the long-standing bandwidth limitations in transformation optics using dual-mode metamaterials that combine Brewster-angle broadband transmission with multiband Fabry-Pérot resonances, enabling broadband invisibility cloaks and flattened retroreflectors.

## INTRODUCTION

The manipulation of electromagnetic wave propagation has emerged as a cornerstone of modern photonic engineering, with metamaterials offering unprecedented control over light–matter interactions [1–6]. A paradigm shift occurred in 2006 with Pendry’s formulation of transformation optics (TO)—a coordinate-transformation framework enabling precise electromagnetic wave manipulation at the level of Maxwell’s equations [7]. This scale-invariant methodology [8–12] has spawned remarkable functional devices, including cloaks [13–18], optical illusion devices [19–22], energy concentrators [23,24], nonlinear optical devices [25], etc. Nevertheless, the extreme material requirements characterized by strong spatial inhomogeneity and anisotropy impose fundamental bandwidth constraints on conventional TO implementations. While theoretical proposals for bandwidth extension exist [26–30], experimental realizations of full-parameter broadband TO devices remain elusive.

To address material complexity challenges, transformation-invariant metamaterials (TIMs) [31]/optic-null media [32,33] were proposed, exploiting singular constitutive parameters (i.e. zero or infinite permittivity/permeability) that remain invariant under arbitrary coordinate transformations. Crucially, their impedance matching with free space enables omnidirectional wave transmission. Practical implementations typically employ subwavelength metallic waveguide arrays operating at either: (i) cutoff frequencies for single-band performance [31,34] or (ii) Fabry–Pérot (FP) resonant frequencies for multiband operation [35–37]. The former approach, while preserving invariance in arbitrary coordinate transformations, suffers from narrowband limitations. The latter, while demonstrating a versatile platform for designing various TO-based devices, such as invisibility cloaks, illusion devices and field concentrators [24], sacrifices transformation invariance through nonzero dielectric constants, restricting applications to uniform coordinate transformations with identical waveguide lengths. This fundamental trade-off has prevented the realization of arbitrarily shaped, multiband TO devices with full transformation invariance.

We present a generalized methodology enabling quasi-broadband TIMs through cascaded impedance-matched metallic slot cavities filled with homogeneous dielectrics. Our architecture achieves dual-functionality: (i) omnidirectional multiband operation and (ii) broadband unidirectional performance, while maintaining transformation invariance under arbitrary coordinate mappings—including those requiring nonuniform propagation paths. Through full-wave simulations and experimental validation, we demonstrate two representative devices: (i) a broadband invisibility cloak exhibiting >88.4% transmittance with minimal phase distortion across 7.5–12.5 GHz, verified through comprehensive near-field and far-field measurements, and (ii) a multiband retroreflector maintaining near-unity retroreflection efficiency in both X and K bands under wide-angle illumination (0$^\circ$–60$^\circ$). This generalized platform resolves the longstanding conflict between operational bandwidth and transformation complexity in TO implementations, enabling the practical realization of arbitrarily shaped metamaterial devices with unprecedented spectral versatility.

## RESULTS

### Theoretical framework of quasi-broadband TIMs

**Figure 1.**
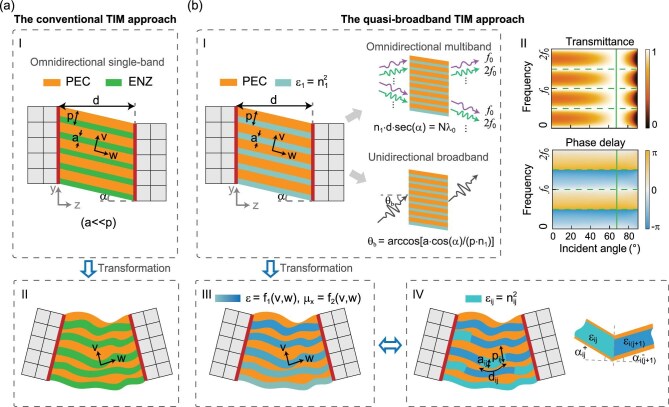
Metamaterial architectures of conventional and dual-mode TIMs. (a) Conventional TIM implementation. (a-I) Cartesian configuration using periodic PEC slots filled with ENZ media; (a-II) transformation-mapped counterpart in curvilinear coordinates maintaining constitutive parameter invariance. (b) Quasi-broadband TIM innovation. (b-I) Base architecture employing PEC slots filled with conventional dielectrics, featuring dual operational modes: Top inset: omnidirectional multiband performance at FP resonance frequencies; bottom inset: unidirectional broadband functionality via Brewster-angle transmission. (b-II) Transmission spectrum (top panel) and phase delay (bottom panel) as a function of the incident angle and of the frequency for the TM waves. The solid line and dashed lines represent the Brewster-angle condition and FP resonance condition, respectively. (b-III) Transformation-mapped implementation requiring inhomogeneous magnetic materials. (b-IV) Practical realization through cascaded impedance-matched dielectric-loaded slot cavities in transformed coordinates, with detailed junction geometry between consecutive segments ($j$, $j + 1$) in channel $i$ shown in magnified view.

Our analysis begins with a description of the fundamental characteristics of TIMs. In Cartesian coordinates, TIMs exhibit singular constitutive parameters expressed as$\ \varepsilon = \mu = \textit{diag}( {1/\infty ,1/\infty ,\infty } )$. This unique parameter set—characterized by infinite and near-zero values—ensures mathematical invariance under coordinate mappings. Moreover, TIMs demonstrate three critical electromagnetic properties: (i) wave propagation strictly confined to the optical axis (aligned with infinite parameters); (ii) null phase accumulation along propagation; and (iii) omnidirectional impedance matching with free space. These properties enable ideal wave transmission through TIM structures without phase distortion or reflection. For transverse magnetic (TM) polarizations, a *w*-axis-oriented TIM can be physically realized through subwavelength periodic structures comprising perfect electric conductor (PEC) slots filled with epsilon-near-zero (ENZ) media (Fig. 1a-I) [31]. To achieve impedance matching, the dielectric thickness-to-period ratio (*a*/*p*) must approach zero ($a,\ p \ll \lambda $), yielding effective parameters: ${\mu }_x \approx 0$, ${\varepsilon }_v \approx 0$ and ${\varepsilon }_w \approx \infty $, which remain invariant under arbitrary coordinate transformations (Fig. 1a-II). While this configuration has enabled experimental implementations of TIMs [31,33,34], its reliance on resonant ENZ media inherently restricts operation to narrow bandwidths.

To overcome this spectral limitation, we developed a hybrid architecture by replacing ENZ media with judiciously cascaded dielectrics (Fig. 1b-IV). The starting point is a modified slot array (Fig. 1b-I) exhibiting effective parameters: ${\mu }_x \approx a/p$, ${\varepsilon }_v = n_1^2p/a$, and ${\varepsilon }_w \approx \infty $ [37], where ${n}_1$ denotes the dielectric refractive index. Consequently, we can derive the analytical reflection (*R*) and transmission (*T*) coefficients when a TM wave is incident on this planar PEC slot array in free space, taking the form:


(1) \begin{equation*}
R = \frac{{\left( {{e}^{ - 2ik^{\prime}_z \, d\sec \alpha } - 1} \right)\left( {{a}^2 {k_0^4}{{\cos }}^2 \alpha - {p}^2 {k_z^2} k^{\prime 2}_z} \right)}} {{{{\left( {ak_0^2 \cos \alpha + p{k}_z {{k^{\prime}_z}}} \right)}}^2 - {e}^{ - 2i{{k^{\prime}_z}}\ d\sec \alpha } {{\left( {ak_0^2 \cos \alpha - p{k}_z {{k^{\prime}_z}}} \right)}}^2}},\end{equation*}

(2) \begin{equation*}
T = \frac{{4ap{e}^{ - i({{k^{\prime}_z}} d\sec \alpha - {k}_y\tan \alpha )}{k}_z{{k^{\prime}_z}} k_0^2\cos \alpha }}{{{{\left( {ak_0^2\cos \alpha + p{k}_z{{k^{\prime}_z}}} \right)}}^2 - {e}^{ - 2i{{k^{\prime}_z}}d\sec \alpha }{{\left( {ak_0^2\cos \alpha - p{k}_z{{k^{\prime}_z}}} \right)}}^2}},\end{equation*}

where ${k}_z = {k}_0 \cos\ \theta $, $k{^{\prime}}_z = {k}_0{n}_1$, $\alpha $ is the tilt angle of the optical axis, $\theta $ is the incident angle and ${k}_0$ is the wave vector in free space. Obviously, omnidirectional impedance matching is maintained when the FP resonance condition is satisfied:


(3) \begin{equation*}
{e}^{ - 2i{{k^{\prime}_z}}d\sec \alpha } = 1 \Rightarrow {n}_1d\sec \left( \alpha \right) = N{\lambda }_0,
\end{equation*}

where ${\lambda }_0$ is the free-space wavelength and $N$ can be any arbitrary integer. This reformulation relaxes the extreme aspect ratio constraint ($a/p \rightarrow 0$) while introducing multiband operation through discrete FP resonances (Fig. 1b-I, top right). Crucially, we identify a complementary broadband mechanism via the Brewster effect [38–41]:


(4) \begin{eqnarray*}
{a}^2 k_0^4 {\cos }^2 \alpha &-& {p}^2 k_z^2 k^{\prime2}_z = 0\nonumber\\
&& \Rightarrow ak_0^2 \cos \alpha -\, p{k}_z {k^{\prime}_z} = 0.
\end{eqnarray*}

By substituting ${k}_z = {k}_0 \cos\ \theta $ and $k{^{\prime}_z} = {k}_0{n}_1$ into Equation (4), total transmission can be achieved at ${\theta }_b = \arccos [ {a\cos \alpha /( {{n}_1p} )} ]$. This non-resonant phenomenon enables broadband performance through optical-axis-aligned propagation with frequency-dependent phase modulation (Fig. 1b-I, bottom right). The synergistic combination of the two mechanisms above establishes a dual-mode TIM platform that fundamentally extends operational bandwidths through discrete FP multiband resonances and Brewster-effect broadband transmission.

A specific example validating these dual operational modes is provided in Fig. 1b-II. The top panel shows the transmission spectra for PEC slot arrays under TM-wave incidence with $\alpha = {0}$$^\circ$, ${n}_1 = 2$, $a/p = 0.8$ and $d = {\lambda }_0/2$. Typical transmission peaks (i.e. 0.5*f*_0_, *f*_0_, 1.5*f*_0_ and 2*f*_0_) arising from FP resonances are marked by dashed lines. These resonances are inherently narrowband but nearly independent of the incidence angle. In contrast, the Brewster-mode transmission occurs at an incident angle of 66.4$^\circ$, exhibiting complete frequency independence while maintaining strong angular selectivity. The phase delay as a function of both the incident angle and the frequency is shown in the bottom panel. The TM wave passes through the PEC slot array with a phase delay of 0 at *f*_0_ and 2*f*_0_, and with a phase delay of –π at 0.5*f*_0_ and 1.5*f*_0_, regardless of the incidence angle. Notably, the frequency dispersion of the phase delay is nearly identical for all incident angles. This uniformity arises because electromagnetic waves, incident at any angle, propagate solely along the optical axis within the structure, thus experiencing identical propagation paths.

The composite system of PEC structures and conventional dielectrics exhibiting these dual transmission mechanisms constitutes the first step of our quasi-broadband TIM implementation. Note that the dielectric-filled configuration depicted in Fig. 1b-I fundamentally breaks transformation invariance under general coordinate mappings owing to its nonzero permittivity. Arbitrary transformations requiring nonuniform propagation paths (Fig. 1b-III) would theoretically demand anisotropic magnetic materials with position-dependent permeability—a requirement introducing both fabrication complexity and significant ohmic losses.

We therefore address the next critical implementation challenge: can inhomogeneous magnetic materials be replaced by conventional dielectrics while preserving quasi-broadband functionality? Our solution emerges through equivalent circuit analysis, revealing that the complex transformed medium (Fig. 1b-III) can be physically realized via cascaded dielectric-loaded slot waveguides (Fig. 1b-IV) when satisfying the generalized impedance-matching condition:


(5) \begin{equation*}\frac{{{a}_{ij}\cos \alpha _{ij}^ + }}{{{p}_{ij}{n}_{ij}}} = \frac{{{a}_{i\left( {j + 1} \right)}\cos \alpha _{i\left( {j + 1} \right)}^ - }}{{{p}_{i\left( {j + 1} \right)}{n}_{i\left( {j + 1} \right)}}},
\end{equation*}

where ${n}_{ij}$, ${d}_{ij}$, ${a}_{ij}$ and ${p}_{ij}$, respectively, denote the refractive index, segment length, dielectric width, and periodicity in the $j$-th segment of the $i$-th channel. Angles $\alpha _{ij}^ - $ and $\alpha _{ij}^ + $ define the optical axis orientation relative to adjacent interfaces (Fig. 1b-IV inset). Detailed proof for the effectiveness of cascaded dielectric-loaded slot waveguides can be found in Data SI.

The system simultaneously satisfies two operational criteria:

(i) Multiband condition (FP resonance):


(6) \begin{equation*}\sum\limits_{j = 1}^m {{n}_{ij} \times {d}_{ij}} = N{\lambda }_0,\end{equation*}

Ensuring equal total optical path lengths ($m$ segments per channel) for omnidirectional multiband operation.

(ii) Broadband condition (Brewster effect):


(7) \begin{equation*}\cos {\theta }_b = \frac{{{a}_{i1}\cos \alpha _{i1}^ - }}{{{p}_{i1}{n}_{i1}}}{\mathrm{ = constant }}\quad \forall i.\end{equation*}

Maintaining channel-independent Brewster angles for ultrabroadband unidirectional transmission.

For applications requiring only multiband functionality, the design constraints simplify to individual FP resonance in each segment:


(8) \begin{equation*}{n}_{ij} \times {d}_{ij} = {N}_{ij}{\lambda }_0.\end{equation*}

This procedure permits arbitrary cascaded architectures through discrete resonant elements, dramatically expanding design flexibility (see Data SII for the detailed proof).

### Implementation of quasi-broadband transformation optical devices

The cascaded architecture satisfying Equations (5–7) establishes a universal platform for implementing arbitrarily shaped TIMs and quasi-broadband TO devices. To validate this paradigm, we consider two typical examples: a full-parameter free-space cloak and an angle-insensitive retroreflector (the design specifications and geometric parameters are detailed in Data SIII).

**Figure 2.**
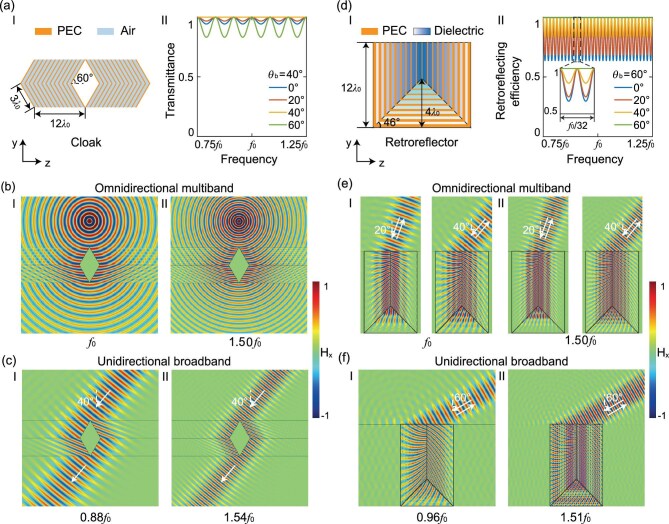
Quasi-broadband TO devices enabled by the cascaded design. (a) Multispectral invisibility cloak: (a-I) Geometric configuration with four TIM sectors (optical axes $ \pm 30^\circ $ tilt); (a-II) Transmittance spectra at different incident angles ${\theta }_i = 0^\circ $, $20^\circ $, $40^\circ $ (the Brewster angle), $60^\circ $. (b) Full-wave verification under point-source illumination at two FP resonances: (I) ${f}_0$ and (II) $1.5{f}_0$. (c) Broadband performance validation via $40^\circ $ Gaussian beam illumination at (I) $0.88{f}_0$ and (II) $1.54{f}_0$. (d) Angle-insensitive retroreflector: (d-I) geometric configuration with three TIM sectors; (d-II) transmittance spectra at different incident angles, i.e. *θ_i_* = 0$^\circ$, 20$^\circ$, 40$^\circ$, 60$^\circ$ (the Brewster angle), respectively. (e) Simulated magnetic field distributions under various spatial Gaussian beams at two FP resonant frequencies (${f}_0$ for Panel I, $1.5{f}_0$ for Panel II) and (f) at two non-resonant frequencies ($0.96{f}_0$ for Panel I, $1.51{f}_0$ for Panel II).

Figure 2a-I illustrates our planar cloak architecture comprising four strategically arranged TIM modules. The upper quadrilateral sectors employ optical axes tilted at ±30$^\circ$ from the vertical, mirrored in the lower sectors. Each sector is constructed by using periodic metallodielectric structures with a uniform propagation length ($3{\lambda }_0$ along the optical axis) and optimized dielectric ratio ($a/p = \cos 40^\circ /\cos 30^\circ $). This configuration enables omnidirectional wave routing around concealed regions at FP resonances as well as broadband cloaking (at ±$40^\circ $ incidence) through Brewster-matched transmission. Figure 2a-II quantifies angular-insensitive performance with a simulated transmittance of >99.96% across all FP resonances (0.75${f}_0$, ${f}_0$, 1.25${f}_0$,…). Full-wave simulations (under point-source illumination) shown in Fig. 2b confirm perfect wavefront reconstruction at two representative resonant frequencies ${f}_0$ (Panel I) and $1.50{f}_0$ (Panel II). Here, we specially highlight the curve for ${\theta }_i=40^\circ $ in Fig. 2a-II, which indicates that, at the Brewster incidence ${\theta }_i = {\theta }_b = \pm 40^\circ $, the cloak exhibits a reflectance of <0.006% over the entire spectrum from $0.75{f}_0$ to $1.25{f}_0$ and beyond. This broadband unidirectional cloaking effect is further verified by using Gaussian beam propagation at two off-resonant frequencies of $0.88{f}_0$ (Fig. 2c-I) and $1.54{f}_0$ (Fig. 2c-II).

The proposed methodology further enables the design of a quasi-broadband retroreflector through the strategic integration of three TIM modules (Fig. 2d-I), i.e. one base module with a horizontally aligned optical axis and two side modules with vertically oriented optical axes. With dimensions optimized to $12{\lambda }_0$ (height) and $8{\lambda }_0/\tan 46^\circ $ (length), the retroreflector incorporates graded PEC slots, each of which consists of three dielectric inserts. These inserts maintain cumulative optical path lengths matching integer multiples of the operational wavelength while compensating for cross-sectional variations between different slots through tailored dielectric filling ratios. This design ensures broadband unidirectional impedance matching at the Brewster angle ${\theta }_b = \pm 60^\circ $. Figure 2d-II presents our simulated results, revealing dual-spectral functionality: angle-independent unitary retroreflection efficiencies at discrete FP resonances coexist with frequency-independent perfect retroreflection across the entire spectral range ($0.75{f}_0-1.25{f}_0$) at ${\theta }_b = \pm 60^\circ $. Full-wave validation demonstrates robust operation through Gaussian beam excitation under diverse conditions: (i) an omnidirectional multiband regime at two FP resonant frequencies ${f}_0$ and $1.50{f}_0$ across a wide range of incident angles (e.g. $20^\circ $ and $40^\circ $, Fig. 2e); (ii) a broadband unidirectional regime from $0.96{f}_0$ to $1.51{f}_0$ at ${\theta }_b = \pm 60^\circ $.

Remarkably, both the cloak and the retroreflector maintain exceptional off-resonance performance, sustaining efficiencies exceeding 83% (cloak) and 64% (retroreflector) at any incident angle. This operational resilience stems from the synergistic combination of resonant phase-matching and non-resonant Brewster transmission mechanisms, establishing a new paradigm for ‘wide-angle broadband TO devices’. This cascaded Brewster transmission mechanism is highly useful and might be used to enhance the efficiency of launchers or extractors for unidirectional guided waves through broadband impedance matching [42] or enable the control of both momentum and orbital angular momentum involving nonplanar interfaces [43].

### Experimental realization

**Figure 3.**
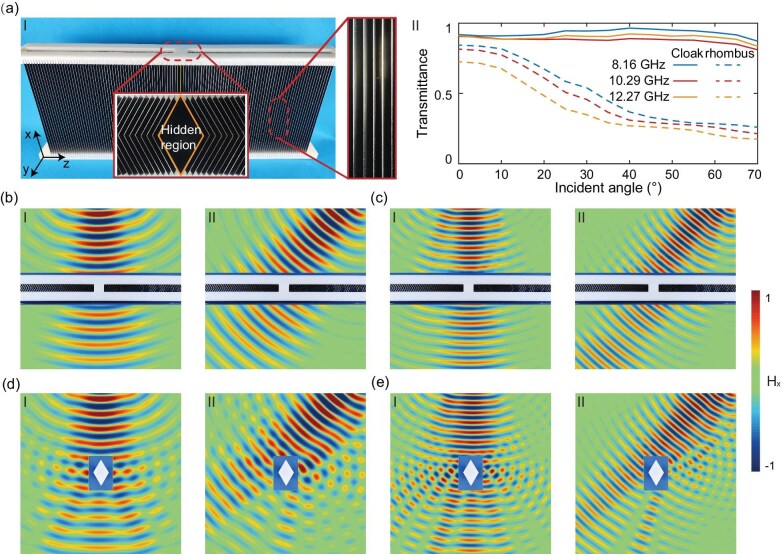
Experimental validation of omnidirectional multiband cloaking performance. (a-I) Fabricated metamaterial cloak and (a-II) angular transmittance spectra comparing cloaked (solid curves) and uncloaked (dashed curves) configurations at three FP resonant frequencies: 8.16 GHz, 10.29 GHz and 12.27 GHz. (b and c) Measured magnetic field distributions for the cloaked system under normal (I: $\theta = 0^\circ $) and oblique (II: $\theta = 40^\circ $) incidence at (b) 8.16 GHz and (c) 12.27 GHz. (d and e) Comparative field perturbations caused by an uncloaked stainless-steel reference object under identical illumination conditions at (d) 8.16 GHz and (e) 12.27 GHz.

We experimentally realize the quasi-broadband cloak through the precise fabrication of its optimized TIM architecture (Fig. 3a-I). The cloak features a total optical path of 75 mm with Brewster-angle operation at ${\theta }_b \approx \pm 40^\circ $ (geometric specifications are detailed in ‘Methods’). The macroscopic structure incorporates stainless-steel metamaterial elements, with insets highlighting the geometry of the concealed region and configurations of the metallic strips. To systematically evaluate the cloaking performance, we conduct comparative microwave measurements in an anechoic chamber by using a reference stainless-steel rhombus matching the dimensions of the concealed object (experimental configurations are detailed in Data SV). Figure 3a-II quantifies the angular performance through transmission-efficiency measurements at three FP resonant frequencies (8.16, 10.29 and 12.27 GHz). The cloak maintains >81% transmittance across incidence angles of $0$$^\circ$–$70 $$^\circ$, outperforming the reference rhombus, which exhibits severe angular-dependent scattering (dashed vs solid curves). It can be observed that the measured transmittances of the proposed cloak increase slightly at ∼40$^\circ$ at all three frequencies, attributed to impedance matching enabled by the Brewster effect. The full-field characterization shown in Fig. 3b and c demonstrates effective wavefront reconstruction at both normal (I) and oblique (II) illumination for two representative resonances (8.16 and 12.27 GHz). In stark contrast, Fig. 3d and e reveals significant near-field distortion caused by the uncloaked rhombus, particularly under oblique incidence.

**Figure 4.**
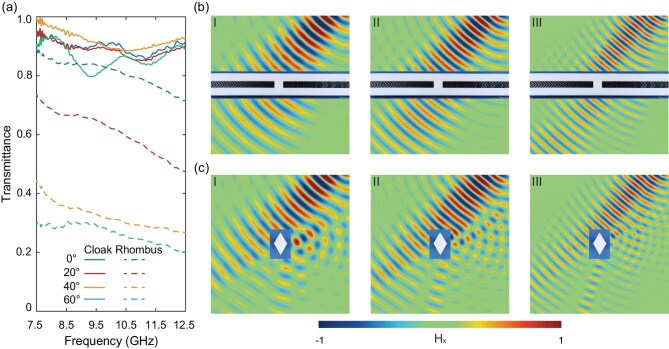
Experimental characterization of quasi-broadband cloaking performance. (a) Angular transmission spectra comparing cloaked (solid curves) and uncloaked (dashed curves) configurations, demonstrating >88.4% transmittance across 7.5–12.5 GHz at ${\theta }_b = 40^\circ $ (Brewster angle) versus angle-limited FP resonances. (b and c) Near-field magnetic field distributions under ${\theta }_b = 40^\circ $ illumination at three representative frequencies: (I) 7.5 GHz, (II) 9.2 GHz and (III) 12.5 GHz. (b) Cloaked system showing preserved wave fronts and (c) uncloaked reference object exhibiting severe scattering.

Figure 4 quantifies the quasi-broadband cloaking performance through angular transmission spectroscopy and near-field measurement. The spectral response shown in Fig. 4a demonstrates frequency-insensitive transmittance of >88.4% across 7.5–12.5 GHz at the Brewster angle ${\theta }_b = 40^\circ $, contrasted with angle-limited FP resonance peaks at other incidence angles. Notably, the transmission efficiency remains at >80% even at off-resonance frequencies, confirming robust broadband functionality. The measured transmittance exhibits frequency-dependent fluctuations, which are likely an artifact of the experimental setup. The non-ideal horn antennas used as the transmitter and receiver possess an impedance mismatch with free space and a significant frequency-dependent response. Consequently, when the sample is placed between them, multiple reflections between the antennas and the sample surfaces occur, inevitably introducing oscillations into the transmittance data. The full-field validation shown in Fig. 4b and c reveals stark performance differences between cloaked and uncloaked configurations under Brewster-angle (${\theta }_b = 40^\circ $) illumination at three representative frequencies: 7.5 GHz (I), 9.2 GHz (II) and 12.5 GHz (III). While the bare stainless-steel rhombus induces severe wavefront distortion at all three frequencies (Fig. 4c), the cloak maintains near-perfect phase reconstruction (Fig. 4b), suppressing >87.6% of the parasitic scattering. These results conclusively establish angle-selective broadband operation through synergistic Brewster transmission and off-resonance impedance matching. Compared with the existing omnidirectional microwave cloak (shown in Table 1), we assert that our design makes a critical advancement in practical transformation optical implementations.

**Table 1.** Comparison of recent work on omnidirectional microwave cloaks.

References	Mechanism	Operating bandwidth
[44]	Metamaterial	Single and narrow band (at 8.5 GHz)
[31]	ENZ media	Single and narrow band (at 4.29 GHz)
[45]		Single and narrow band (at 10 GHz)
[34]		Single and narrow band (at 5 GHz)
[36]	Metamaterial and FP media	Single and narrow band (at 8.0 GHz)
[37]	FP media	Multiple and narrow band (at 5 and 10 GHz)
This work	Dual-mode metamaterial	Quasi-broadband (over 7.5–12.5 GHz)

**Figure 5.**
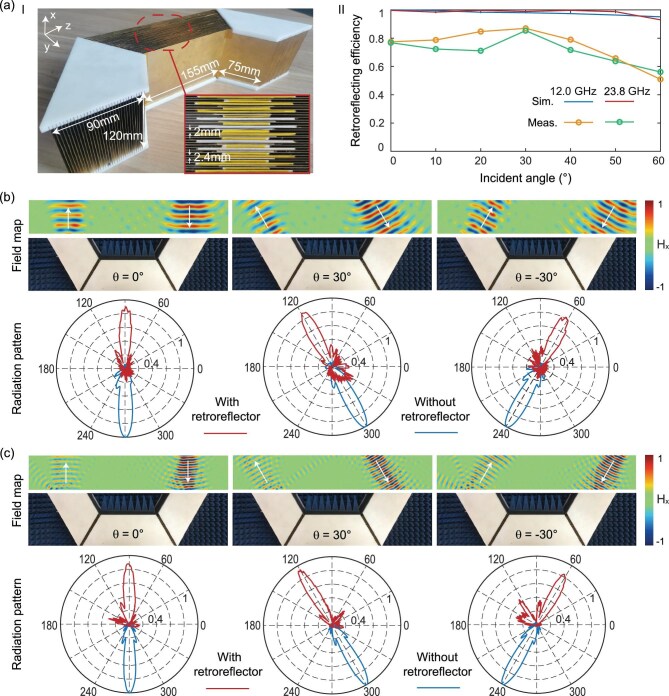
Experimental characterization of the quasi-broadband retroreflector. (a-I) Fabricated prototype and (a-II) angular retroreflection efficiency spectra demonstrating >50% retroreflection efficiency at FP resonances (e.g. 12.0, 23.8 GHz) across 0$^\circ$–60$^\circ$ incidence. (b and c) Multimodal field analysis at (b) 12.0 GHz and (c) 23.8 GHz. Top panels: near-field magnetic distributions for $\theta \ = \ 0^\circ $, $ \pm 30^\circ $ illumination; bottom panels: corresponding far-field radiation patterns with and without the retroreflector captured by the horn antenna.

We next experimentally validate the quasi-broadband retroreflector. Figure 5a-I displays the fabricated prototype with optimized geometry. The structure employs uniform 120$^\circ$-bent copper slots and Teflon dielectric inserts of graded thicknesses to ensure wavelength-multiplied optical paths (inset, Fig. 5a-I), with full geometric parameters and simulation benchmarks provided in Data SIV. Details of the experimental setup are given in Data SV. Angular retroreflection efficiency measurements (Fig. 5a-II) demonstrate >50% performance across 0$^\circ$–60$^\circ$ incidence at two FP resonances (12.0 and 23.8 GHz), though minor deviations from the simulated results arise from fabrication imperfections and experimental misalignments. To enhance the measured retroreflection efficiency of the fabricated sample, there are several potential strategies, including utilizing low-loss, low-dispersion dielectric fillings, shortening the total length of each metallic slot, reducing the arrangement period of the metallic slots, employing more precise manufacturing techniques and increasing the aperture of the fabricated sample.

Full-wave measurement combines near-field imaging and far-field pattern analysis (Fig. 5b and c). At 12.0 GHz, near-field mappings (Fig. 5b, top panels) reveal minimal input reflection and phase-preserved backward radiation for $\theta \ = 0^\circ $ and $ \pm 30^\circ $ illumination. Quantitative far-field profiles (Fig. 5b, bottom panels) yield retroreflection efficiencies of 80.8% ($\theta \ = 0^\circ $), 79.4% ($\theta \ = + 30^\circ $) and 66.4% ($\theta \ = - 30^\circ $), with angular deviations of $ \le 3^\circ \ $from the theoretical predictions. Parallel measurements at 23.8 GHz (Fig. 5c) show comparable performance (77.4%–87.2% efficiency and $ \le 1^\circ $ deviation), albeit with slight frequency shifts owing to thickness variations of the dielectric insert. The observed efficiency reduction at higher frequencies stems from parasitic phase accumulation errors (i.e. the total phase accumulations in some slots deviate from integer multiples of $2\pi $) caused by inaccuracies of the subwavelength Teflon dimensions, manifesting as secondary lobes in the radiation patterns (see Data SVI for a detailed analysis of fabrication tolerances).

The performance of the retroreflector exhibits significant scalability through two complementary strategies: aperture expansion and local resonance tuning. Strategically increasing the unit cell periodicity (via slot array scaling) enhances angular acceptance while maintaining phase coherence across the whole structure—a critical factor for improving wide-angle retroreflection efficiency. Concurrently, dynamic frequency reconfiguration can be achieved through precision adjustments of the Teflon insert dimensions within individual slots, enabling phase compensation without structural redesign. Data SVII validates this procedure through a reconfigured prototype at 15.0 GHz, demonstrating negligible efficiency degradation across full angular coverage compared with the baseline design. This hybrid scaling-tuning approach establishes a versatile platform for adaptive TO devices capable of balancing spectral agility with fabrication practicality.

## CONCLUSION

We present a universal framework for quasi-broadband TO by leveraging cascaded impedance-matched metallic cavities that emulate the essential properties of TIMs. This approach eliminates the need for extreme constitutive parameters while enabling dual-spectral functionality: omnidirectional multiband operation via FP resonance and broadband unidirectional performance through Brewster-angle transmission. By implementing dielectric-filled metallic slot arrays, we significantly simplify the fabrication of arbitrarily shaped TO devices and enable dynamic frequency reconfiguration through local structural tuning. Experimental validation with two prototypes—a quasi-broadband cloak and a multiband retroreflector—demonstrates the versatility of this approach. The cloak achieves >87.6% scattering suppression across 8.0–12.0 GHz (X band) under wide-angle illumination (0$^\circ$–70$^\circ$), alongside wideband >88.4% transmittance at ${\theta }_b = 40 $$^\circ$ spanning 7.5–12.5 GHz. Simultaneously, the retroreflector maintains 66%–87% efficiency in both X and K bands (12.0–23.8 GHz) with ±30$^\circ$ angular tolerance. The modular architecture further supports performance scalability: aperture expansion enhances the angular coverage, while subwavelength dielectric adjustments enable frequency agility. Although experimentally verified at microwave frequencies, the methodology is fundamentally extensible to sub-terahertz regimes in which metallic conductivity remains favorable (see Data SVIII for detailed demonstrations). Furthermore, our current work is essentially confined to 2D configurations and operates exclusively for TM-polarized waves. To circumvent this polarization dependence, a promising strategy involves infiltrating the holey perfect conductor films, proposed by Pendry *et al.*, with anisotropic materials possessing high permittivity and permeability [46]. While such a structure can readily support polarization-independent FP resonances, achieving the simultaneous polarization-insensitive and broadband Brewster effect remains a significant challenge. Certainly, the future availability of broadband, low-dispersive and customizable magnetic materials would make the extension of our design to dual-polarization and full 3D implementations entirely feasible. Additionally, incorporating specialized metasurface designs could extend our method to other polarization states, such as elliptical polarization [47]. This work bridges theoretical TO concepts with practical photonic engineering, opening avenues for next-generation devices such as diffraction-limited superlenses, non-reciprocal waveguides and adaptive beam-steering systems—all achievable through scalable nanofabrication techniques.

## METHODS

The quasi-broadband cloak is composed of 240 identical iron sheets (37.5 mm in length, 210 mm in height and 0.4 mm in thickness). All metal sheets are fixed by two 3D-printed nylon coverers with specifically distributed grooves (with a separation of 4 mm) on the surface. Similarly, the multiband retroreflector consists of 96 copper sheets, which have uniform thickness and height of 0.4 mm and 120 mm, but various lengths (see Data SIV for details). The Teflon sheets with various thicknesses and lengths are glued closely in the middle copper sheets of the retroreflector. In the end, two Nylon bases with specifically distributed grooves (with a separation of 2 mm) were fabricated to hold the composite of copper sheets and Teflon sheets firmly.

## Supplementary Material

nwag023_Supplemental_File
