# Supplementary material for: Breaking bandwidth limits in transformation optics with Brewster-enhanced metamaterials
Source: Natl Sci Rev. 2026 Jan 14;13(5):nwag023. doi: 10.1093/nsr/nwag023 (PMC12976604; doi:10.1093/nsr/nwag023)
Supplement: nwag023_Supplemental_File [file nwag023_supplemental_file.docx]

Supplementary Data

Breaking bandwidth limits in transformation optics with Brewster-enhanced metamaterials

**Author**

Xiaojun Hu^1^, Yu Luo ^2^*, Jingxin Tang^1^, Chun Wang^1^, Yuan Gao^1,3^, Jingjing Zhang^4, 5^*, Yi Zhang^1^, and Dexin Ye^1^*

**Affiliations**

^1^Laboratory of Applied Research on Electromagnetics (ARE), Zhejiang University, Hangzhou 310027, China.

^2^National Key Laboratory of Microwave Photonics, Nanjing University of Aeronautics and Astronautics, Nanjing 211106, China.

^3^School of Electrical and Electronic Engineering, Shandong University of Technology, Zibo 255000, China

^4^State Key Laboratory of Millimeter Waves, Southeast University, Nanjing 210096, China.

^5^Institute of Electromagnetic Space, Southeast University, Nanjing 210096, China.

*Corresponding authors. E-mails: yu.luo@nuaa.edu.cn; zhangjingjing@seu.edu.cn; desy@zju.edu.cn.

**I. Performance of the cascaded impedance-matched** **dielectric-loaded slot waveguides**

In this section, we will show that the cascaded dielectric-loaded slot waveguides can be equivalent to the slot filled with a single magnetic dielectric.


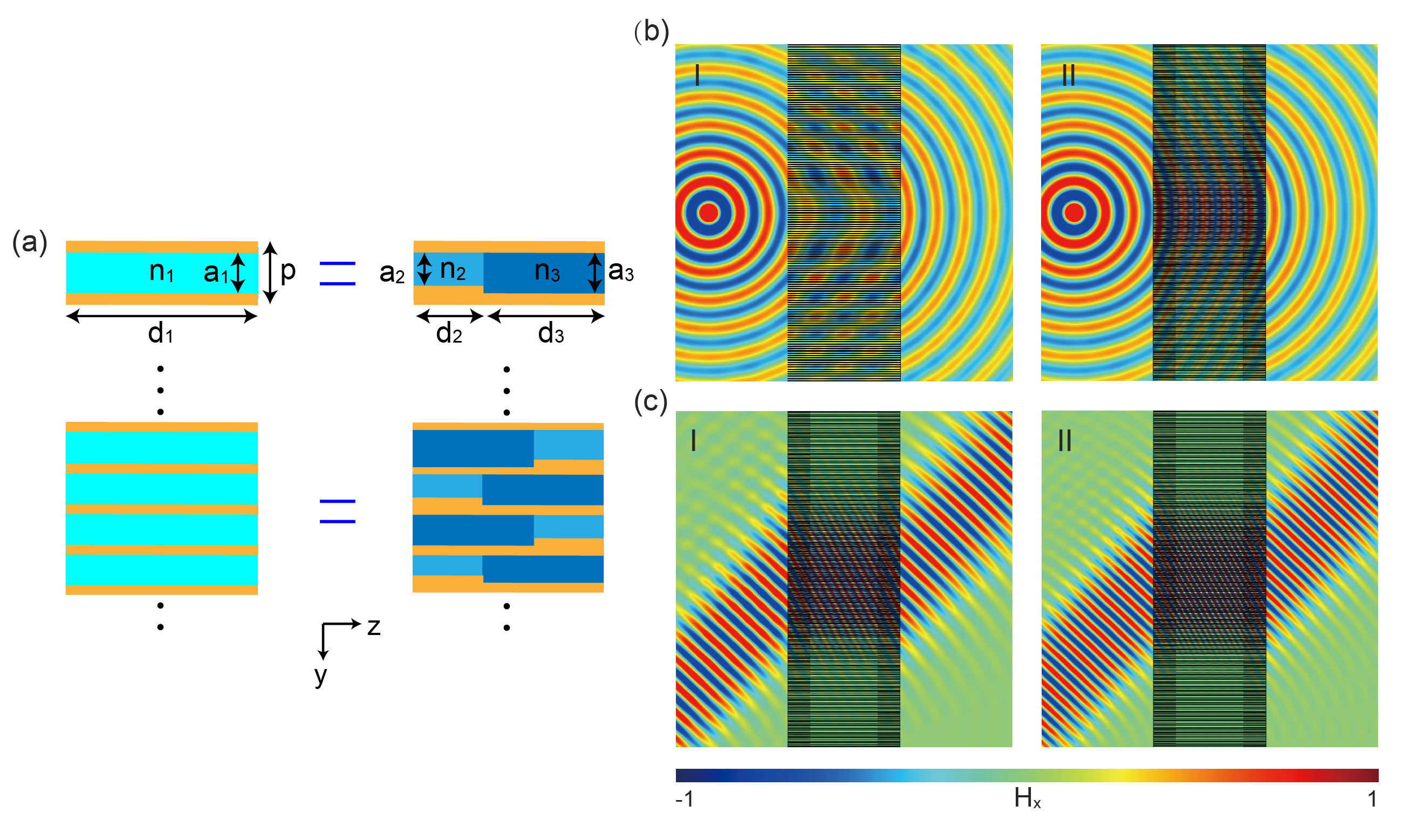


**Figure S1.** Performance of the cascaded dielectric-loaded slot waveguides. (a) Schematic diagram of the equivalence between the PEC slot filled with a magnetic dielectric and cascaded impedance-matched PEC slots filled with normal dielectrics. (b) Simulated magnetic field distributions while placing a point source with an *x*-polarized magnetic field in front of the PEC slot arrays filled with a single magnetic dielectric (I) and cascaded impedance-matched PEC slots filled with normal dielectrics (II). (c) Simulated magnetic field distributions of cascaded impedance-matched PEC slot array under Gaussian beam incidence at two non-resonant frequencies, ${1.45f}_{0}$ (I) and ${1.90f}_{0}$(II).

The left panel of Fig. S1a shows a PEC slot with a length of $d_{1}$, composed of two parallel PEC strips with a subwavelength distance and a magnetic material (with a refractive index $n_{1}$). According to Eq. 3 in the main text, the waves at the two ends of the slot have equal amplitude and phase at the Fabry-Pérot (FP) resonant frequencies ($n_{1}d_{1}=N_{1}\lambda_{0}$, $N_{1}$ can be any arbitrary integer). Moreover, due to its subwavelength cross-section, only transverse electromagnetic (TEM) mode exists inside the slot. With [this](https://cn.bing.com/dict/search?q=this&FORM=BDVSP6&cc=cn) [property](https://cn.bing.com/dict/search?q=property&FORM=BDVSP6&cc=cn)[,](https://cn.bing.com/dict/search?q=%2C&FORM=BDVSP6&cc=cn) the PEC slots will be equivalent to each other while replacing the single magnetic dielectric with two (or more) cascaded normal dielectrics with various occupancies, only if they satisfy $n_{2}d_{2}+n_{3}d_{3}=N_{2}\lambda_{0}$ and $n_{2}a_{3}=n_{3}a_{2}$, as shown in the right panel of Fig. S1a. Consequently, we can assert that the PEC slot array filled with a magnetic dielectric is also equivalent to the cascaded impedance-matched PEC slots filled with normal dielectrics, as depicted in the bottom panels of Fig. S1a. The only requirement is that two adjacent cascaded slot segments are impedance-matched to each other, and the total optical lengths of all the segments satisfy the FP condition.

As an example, Fig. S1b shows the simulated magnetic field distributions while placing a point source with an *x*-polarized magnetic field in front of two FP resonant PEC slot arrays. All slots in panel I are filled with a magnetic dielectric with $n_{1}=0.5$ ($d_{1}=4\lambda_{0}$), while those in panel II are filled with two cascaded normal materials with $n_{2}=1$ ($d_{2}=0.8\lambda_{0}$) and $n_{3}=21/16$ ($d_{3}=3.2\lambda_{0}$). The orders of two dielectrics in the adjacent two slots are changed intentionally to show universality. In the former case, the width of the dielectric $a_{1}$ is ${9\lambda}_{0}/400$ and the period of the unit cell $p$ is $\lambda_{0}/40$. While in the later one, the widths of the dielectric $a_{2}$ and $a_{3}$ are respectively ${3\lambda}_{0}/175$ and ${9\lambda}_{0}/400$ and the period of the unit cell $p$ is $\lambda_{0}/40$. We can see that the magnetic field distributions outside two slot arrays are nearly the same, denoting that the FP resonant PEC slots filled with a single magnetic dielectric can be equivalent to the cascaded impedance-matched PEC slots filled with normal dielectrics. Moreover, according to the Eq. 4 in the main text, ultra-broadband perfect transmission occurs under incident angles of ± $46.7^{\circ}$ in the cascaded impedance-matched PEC slots. Fig. S1c shows the simulated magnetic field distributions under incidence angle of $46.7^{\circ}$ at two non-resonant frequencies: ${1.45f}_{0}$(I) and ${1.90f}_{0}$(II). It is seen that near-perfect transmittance occurs, demonstrating quasi-broadband behavior.

**II. Transformation-invariance of cascaded dielectric-loaded slot waveguides**

In this section, we quantitatively demonstrate the transformation-invariance of our design. We have performed a detailed analysis of a waveguide with non-uniform optical-path mappings, as schematically illustrated in Fig. S2a. The waveguide has a different physical length for each channel, and each cascaded, dielectric-loaded slot within a channel is designed to be impedance-matched to its neighbors (satisfying Eq. 5 in the main text). The detailed geometric parameters (listed in Table S1) result in a total optical path length of 150 mm and a Brewster angle of 60°.


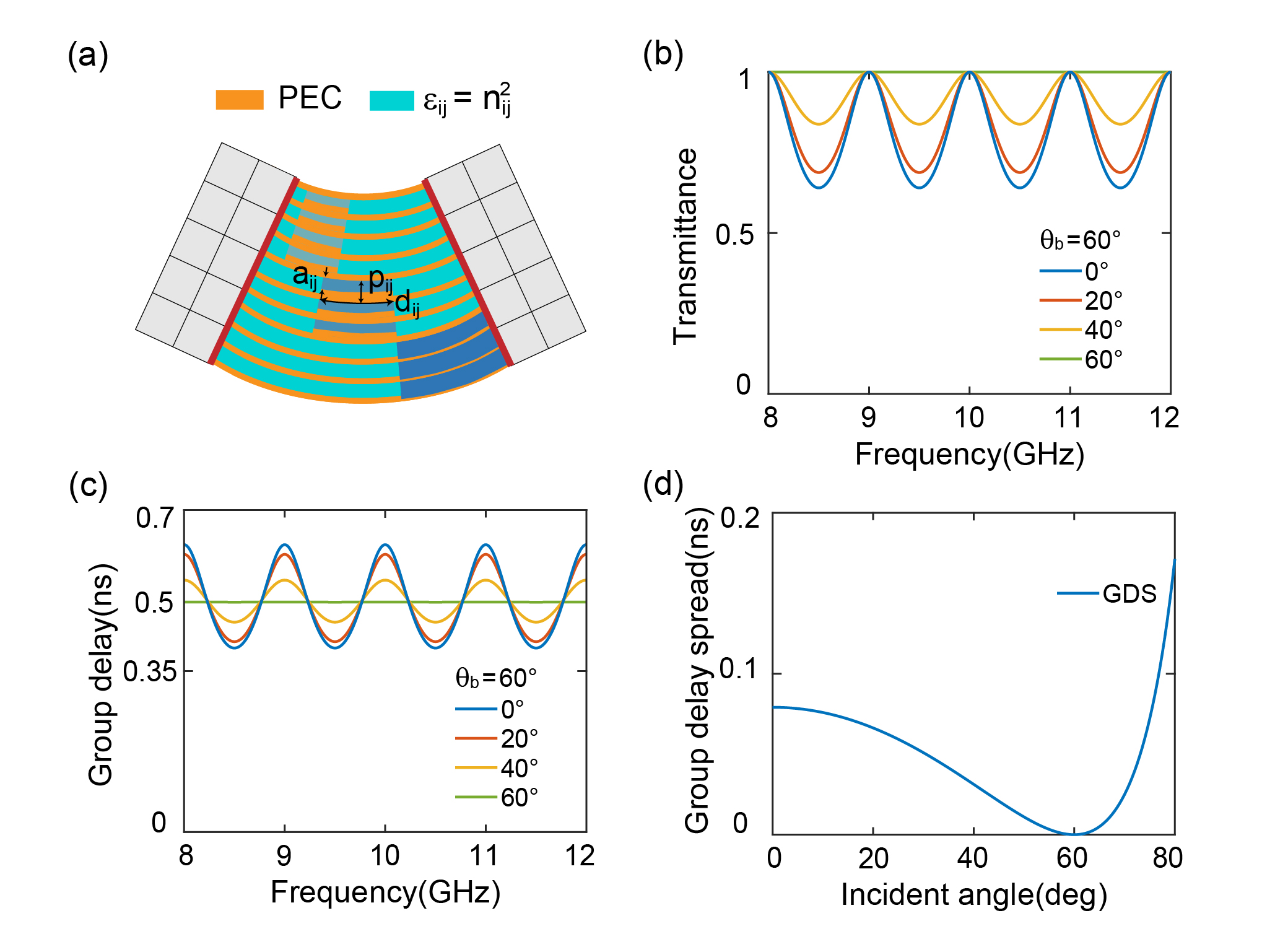


**Figure S2.** Transformation‑invariance under non‑uniform optical‑path mappings. (a) Schematic of cascaded impedance-matched dielectric-loaded slot waveguides. (b) Transmittance spectra at different incident angles *θ_i_* = 0° (blue), 20° (red), 40° (orange), 60° (green, the Brewster angle). (c) Angular group delay spectra. (d) Group delay spread as a function of the incidence angle.

The simulated transmittance, shown in Fig. S2b, demonstrates that the structure exhibits near-unity transmission across the entire X-band at the Brewster angle $\theta_{b}=\pm60^{\circ}$. At other angles, distinct FP resonant peaks are clearly visible. The group delays ($\tau g$), shown in Fig. S2c, are calculated by $\tau g\left( \omega\right)=-d\varphi(\omega)/d\omega$, where $\varphi$ is the phase delay. When TM waves are incident at the Brewster angle, the group delay is near constant (0.5 ns) across the entire operational band. This value is precisely the time required for light to travel the total optical path length of 150 mm, demonstrating that all spectral components transmit without temporal distortion. In contrast, for other incidence angles, the group delay varies significantly with frequency. To quantify this effect, we define the group delay spread (GDS) as the standard deviation of the group delay, as illustrated in Fig. S2d. We observe that GDS is near zero at the Brewster angle, quantitatively confirming the absence of group delay dispersion.

| *Sequence* | *Total length l_n_ (mm)* | *Filling length d_n_ (mm)* | *The permittivity of the filling dielectric ε_r_n_* | *Duty cycle of dielectric a_n_/p_n_* |
| --- | --- | --- | --- | --- |
| *1* | 104.7 | 52.4 | 3.48 | 0.93 |
| *2* | 107.3 | 53.7 | 3.22 | 0.90 |
| *3* | 110.0 | 55.0 | 2.98 | 0.86 |
| *4* | 112.6 | 56.3 | 2.78 | 0.83 |
| *5* | 115.2 | 57.6 | 2.57 | 0.80 |
| *6* | 117.8 | 58.9 | 2.39 | 0.77 |
| *7* | 120.0 | 60.2 | 2.22 | 0.75 |
| *8* | 123.0 | 61.5 | 2.07 | 0.72 |
| *9* | 125.66 | 62.8 | 1.92 | 0.69 |
| *10* | 128.28 | 64.1 | 1.79 | 0.67 |

**Table S1.** Geometric parameters of the waveguide and the dielectric-loaded segments. The remaining segments are air-filled with a duty cycle of 0.5.

**III. Designs of the quasi-broadband cloak and retroreflector**

In this section, we show the detailed designs of the cloak and the retroreflector and their constitutive parameters, corresponding to Fig. 2 in the main text.


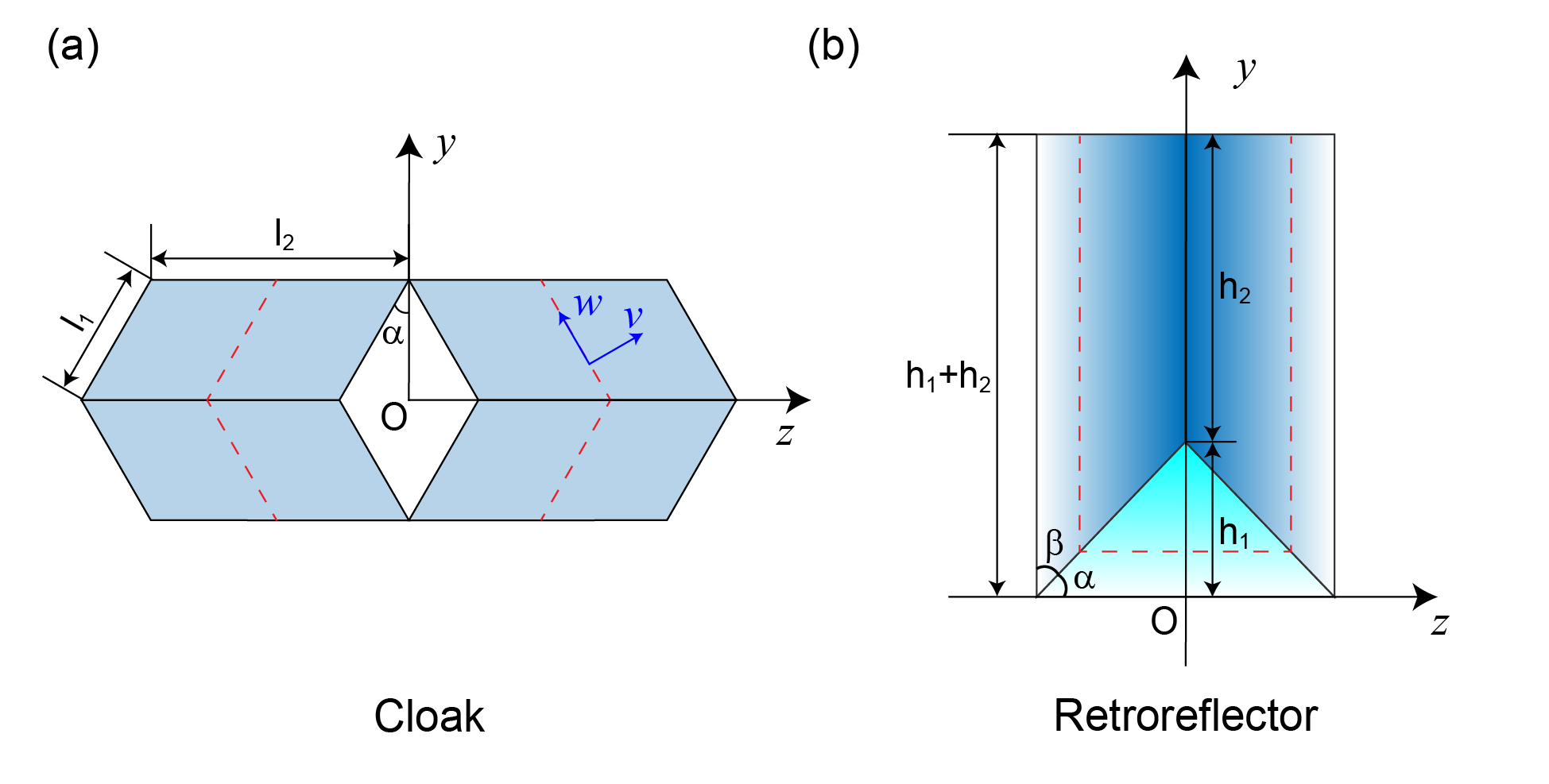


**Figure S3.** Schematic diagram of quasi-broadband cloak and retroreflector. (a) Schematic diagram of the cloak composed of homogeneous regions. (b) Schematic diagram of the retroreflector composed of cascaded impedance-matched inhomogeneous regions.

Fig. S3a shows the schematic diagram of the designed cloak. This cloak consists of four homogeneous regions judiciously glued together. The homogeneous regions are parallelograms with length of ${l_{1}=3\lambda}_{0}$ and ${l_{2}=12\lambda}_{0}$ and their constitutive parameters are $\varepsilon_{w}\approx\infty$, $\varepsilon_{v}=1/0.88$, $\mu_{x}\approx0.88$. The dashed red lines mark the corresponding optical axes, which are oriented with angles of $\alpha=\pm30^{\circ}$ from the *y*-axis. Such a design guides the incident waves to travel around the cloaked region omnidirectionally at FP resonant frequencies and unidirectionally at ultra-broadband frequency.

Fig. S3b shows the schematic diagram of quasi-broadband retroreflector, consisting of three inhomogeneous regions. The dashed red lines mark the optical axes of three regions, from which we can see that the optical axis of the TIM at the bottom orientates along the horizontal direction, whilst the optical axes of the top TIMs align along the vertical direction. The dimensions of the retroreflector are ${h_{1}=4\lambda}_{0}$, ${h_{2}=8\lambda}_{0}$, and the angles are $\alpha=46^{\circ}$, $\beta=44^{\circ}$. According to the Eqs. 6-7 in the main text, quasi-broadband behavior requires that the constitutive parameters of three regions satisfy:

$$\varepsilon_{y}=\infty,\varepsilon_{z}=\frac{N\lambda_{0}}{\left(2\cdot\left(\left|x\right|\tan\alpha+h_{2}\right)+2\cdot\left|x\right|\right)\cdot\cos\theta_{b}},\mu_{x}=\frac{N\lambda_{0}\cos\theta_{b}}{\left(2\cdot\left(\left|x\right|\tan\alpha+h_{2}\right)+2\cdot\left|x\right|\right)}$$

for top regions, (S1)

$$\varepsilon_{y}=\frac{N\lambda_{0}}{\left(2\left(h_{1}-y\right)\tan\beta+2\left(h_{1}+h_{2}-y\right)\right)\cdot k\cdot\cos\theta_{b}},\varepsilon_{z}=\infty,\mu_{x}=\frac{kN\lambda_{0}\cos\theta_{b}}{2\left(h_{1}-y\right)\tan\beta+2\left(h_{1}+h_{2}-y\right)}$$

for bottom region, (S2)

where $N=32$, $\theta_{b}$ is the Brewster angle and $k=sin \beta/sin\alpha$, which represents the cross-section ratio between the top segments and bottom segment. By substituting ${h_{1}=4\lambda}_{0}$, ${h_{2}=8\lambda}_{0}$ and $\theta_{b}=60^{\circ}$ into Eqs. S1-S2, we can conclude that the three regions are not involved with magnetic materials and can be realized by cascaded PEC slots with judiciously chosen normal materials with various occupancies.

**IV. Designed retroreflector operating at 12 GHz**

In this section, we show the detailed designs of the multiband retroreflector, corresponding to Fig. 5 in the main text. As shown in Fig. S4a, for ease of physical implementation, the bending angles of all copper slots are set to be $120^{\circ}$ so that all the slots have a uniform cross section. Such a design also ensures that the input and output surfaces are sufficiently far from each other to distinguish the output retroreflected beam from the input one. Considering the actual manufacture, the thickness of copper strips is chosen to be 0.4 mm, the thickness of the slot is 2 mm ($w$ = 2 mm), and the operating frequencies are set at 12 GHz and 24 GHz. In the actual design, 32 bent copper strips are used and their lengths $a_{n}$ form an arithmetic series which, from the bottom to the top, satisfies $a_{n}=a_{1}-(n-1)\times d_{s}$ ($n=1, 2, 3 ... 32$), where $a_{1}=560 mm$ and $d_{s}=8.31 mm$. Sandwiched between these 32 copper strips are 31 slot waveguides, whose lengths $l_{n}[l_{n}={(a}_{n}+a_{n+1})/2,n=1, 2, 3, ... 31]$also form an arithmetic series, as given in Table S2.


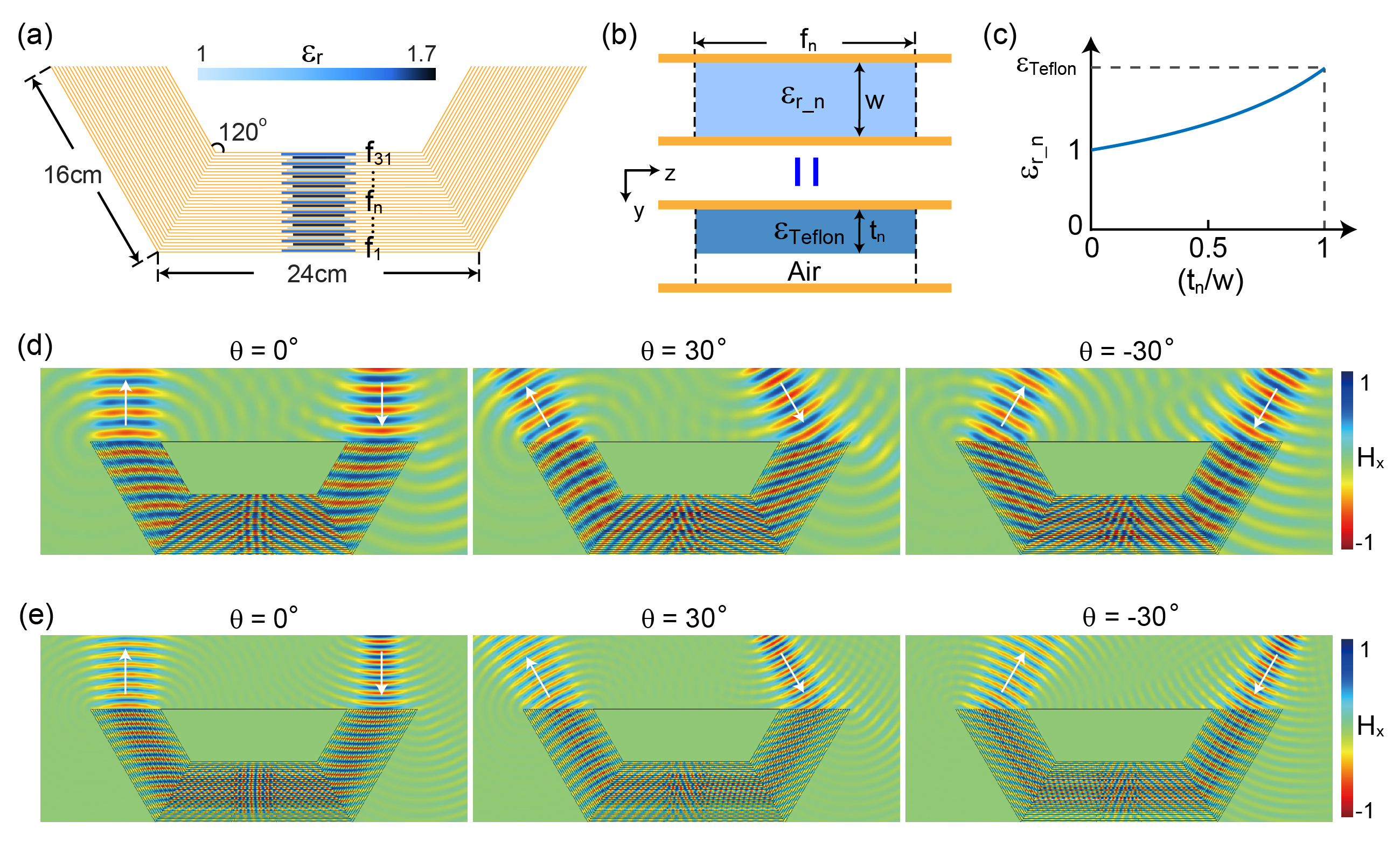


**Figure S4.** Actual design of the retroreflector and full-wave simulation. (a) Designed retroreflector consisting of copper strips and Teflon sheets. (b) Realization of desired dielectrics using thin Teflon sheet and air gap based on the effective medium theorem, and (c) its effective permittivity with respect to the filling thickness of Teflon. Simulated magnetic field distributions under different incident angles ($0^{\circ}$, $30^{\circ}$, -$30^{\circ}$) at 12 GHz (d) and 24 GHz (e).

To ensure that the total propagation length in each slot is a multiple of the wavelength, normal dielectrics are filled in the center of each slot, as denoted by the blue regions in Fig. S4a. In this case, the two air slots on the left and right of the material are identical and their lengths can be set as a multiple of the half-wavelength. The dielectric length $f_{n}$ ($n=1, 2, 3 ... 31$) of each slot is given in Table S2. Note that the permittivities of all filling dielectrics are smaller than that of Teflon ($\varepsilon_{teflon}=2.01$), but larger than unity. They can thus be effectively realized by filling each slot with Teflon sheets of different thicknesses [1], as shown in Fig. S4b. Since the electric field in each slot is polarized along the *y* direction, the effective dielectric permittivity $\varepsilon_{r\_n}$ is obtained as:

$\varepsilon_{r\_n}=\frac{w\varepsilon_{teflon}}{\left(w-t_{n}\right)\varepsilon_{teflon}+t_{n}}$, (S3)

where $w$ is the slot width and $t_{n}$ denotes the thickness of the Teflon sheet in the $n_{th}$ slot. Fig. S4c plots $\varepsilon_{r\_n}$ as a function of the ratio $t_{n}/w$ The thicknesses of the Teflon sheets $t_{n}$ and corresponding effective permittivities $\varepsilon_{r\_n}$ for all 31 slots are provided in Table S2.

| *Sequence* | *Length of copper strips a_n_ (mm)* | *Filling length f_n_ (mm)* | *The permittivity of the filling dielectric ε_r_n_* | *Filling thickness of Teflon t_n_ (mm)* |
| --- | --- | --- | --- | --- |
| *1* | 560.00 | 80.15 | 1.56 | 1.40 |
| *2* | 551.69 | 46.84 | 1.14 | 0.47 |
| *3* | 543.38 | 38.52 | 1.68 | 1.60 |
| *4* | 535.07 | 80.21 | 1.55 | 1.40 |
| *5* | 526.73 | 46.90 | 1.14 | 0.46 |
| *6* | 518.42 | 38.58 | 1.68 | 1.59 |
| *7* | 510.11 | 80.27 | 1.55 | 1.40 |
| *8* | 501.80 | 46.95 | 1.13 | 0.46 |
| *9* | 493.49 | 38.64 | 1.67 | 1.58 |
| *10* | 485.18 | 80.33 | 1.55 | 1.39 |
| *11* | 476.87 | 47.01 | 1.13 | 0.45 |
| *12* | 468.56 | 38.70 | 1.67 | 1.58 |
| *13* | 460.22 | 80.38 | 1.55 | 1.38 |
| *14* | 451.91 | 47.07 | 1.13 | 0.44 |
| *15* | 443.60 | 38.76 | 1.66 | 1.57 |
| *16* | 435.29 | 80.44 | 1.55 | 1.38 |
| *17* | 426.98 | 47.13 | 1.13 | 0.43 |
| *18* | 418.67 | 38.82 | 1.66 | 1.56 |
| *19* | 410.36 | 80.50 | 1.54 | 1.37 |
| *20* | 402.05 | 47.19 | 1.12 | 0.43 |
| *21* | 393.71 | 38.87 | 1.65 | 1.56 |
| *22* | 385.40 | 80.56 | 1.54 | 1.37 |
| *23* | 377.09 | 47.25 | 1.12 | 0.42 |
| *24* | 368.78 | 38.93 | 1.65 | 1.55 |
| *25* | 360.47 | 80.62 | 1.54 | 1.37 |
| *26* | 352.16 | 47.30 | 1.12 | 0.41 |
| *27* | 343.85 | 38.99 | 1.64 | 1.54 |
| *28* | 335.54 | 80.68 | 1.54 | 1.36 |
| *29* | 327.20 | 47.36 | 1.11 | 0.40 |
| *30* | 318.89 | 39.05 | 1.64 | 1.53 |
| *31* | 310.58 | 80.71 | 1.53 | 1.36 |
| *32* | 302.27 | None | None | None |

**Table S2.** Geometric details of the copper strips and the filling Teflon sheets.

To verify the performance of the design above, we perform full-wave simulations on the actual structure (i.e., the retroreflector consisting of only copper strips and Teflon sheets) using commercial software COMSOL Multiphysics. Limited to the aperture size, we only take $\pm30^{\circ}$ as the oblique incident angle. To reduce the interference from the incident Gaussian beam, the trapezoidal region in front of the retroreflector is filled with perfectly matched absorbers. Figs. S4d and S4e show the simulated magnetic field distributions for three incident angles (*θ* = $0^{\circ}$, $30^{\circ}$, and $-30^{\circ}$) at 12 GHz and 24 GHz, respectively. In each subfigure, the right and left white arrows denote the directions of the incident beam and the retroreflected one, respectively. As we expected, no obvious reflection is observed at the incident surface in all three cases, implying perfect impedance matching and high retroreflecting efficiencies.

**V. Details of the experimental setups**

In this section, we show the details of the experimental setups and the measurement procedures, corresponding to the results of Figs. 3-5 in the main text. All the measurements were characterized in an anechoic chamber using an Agilent 8722ES network analyzer. In the measurement of transmittance for the cloak, two identical horn antennas, respectively served as transmitter and receiver, were placed on the two sides of the cloak with a distance of $L_{1}=0.5 m$, as shown in Fig. S5a. The transmittance was then measured by rotating the horn antennas from 0 to $70^{\circ}$.


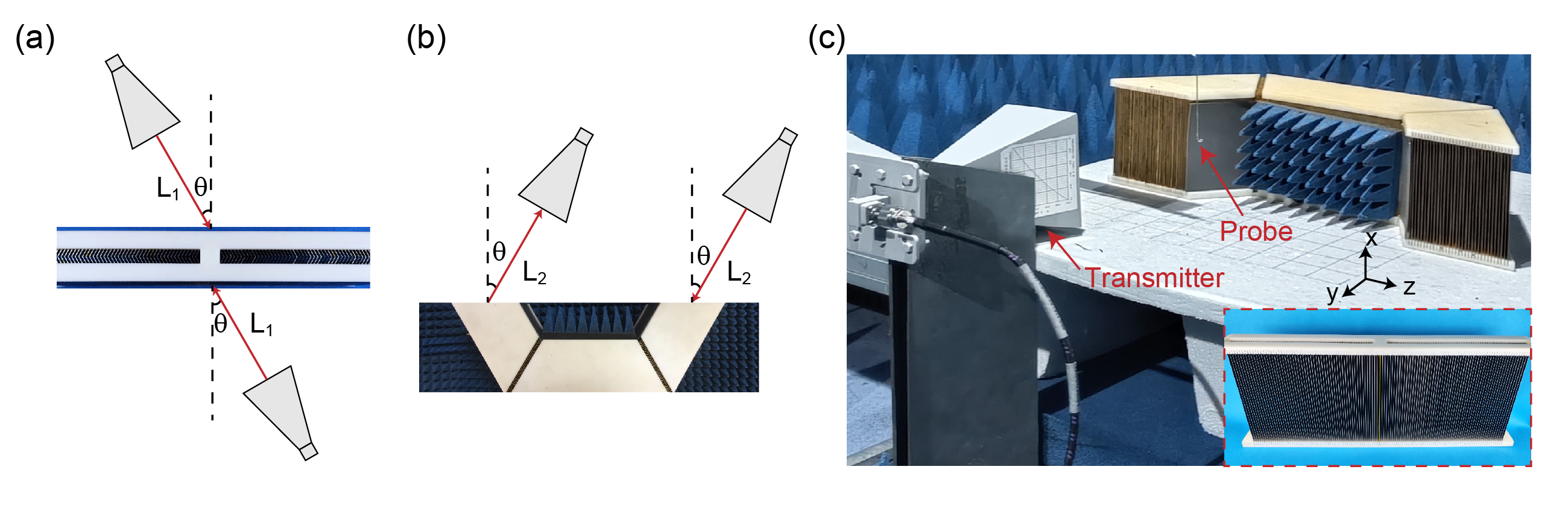


**Figure S5. Experimental setup.** Schematic of the experimental setup in measuring transmittance (a) and retroreflecting efficiency (b). (c) Photograph of the experimental setup for the near-field distribution measurement.

Similarly, in the measurement of retroreflecting efficiency, two identical horn antennas were placed in front of two ports with a distance of $L_{2}=0.2 m$, as shown in Fig. S5b. The retroreflecting efficiency was measured by rotating the horn antennas from 0 to $60^{\circ}$. Fig. S5c shows the photograph of experimental setup for near-field distribution measurement, which is identical to the cloak and retroreflector. A horn antenna was used to excite the TM wave incidence, and a homemade loop antenna, controlled by a mechanical arm, was used as the probe to detect the magnetic field point-to-point to obtain the near-field distributions. The field-scanning areas of the cloak are 400 mm × 400 mm with a spatial resolution of 4 mm. The field-scanning area in front of the retroreflector is 522 mm × 90 mm, and the spatial resolutions for 12 GHz and 23.8 GHz are 3 mm and 1.5 mm, respectively. All the perimeters of the homemade loop antenna probes are half of the operating wavelength.

**VI. Analysis of the Fabrication Tolerances**

In this section, we analyze the sensitivity of our design to fabrication tolerances. Theoretical considerations regarding the FP resonance and the Brewster effect suggest that the refractive index ($n$) acts as the most critical parameter governing the system's performance.

*
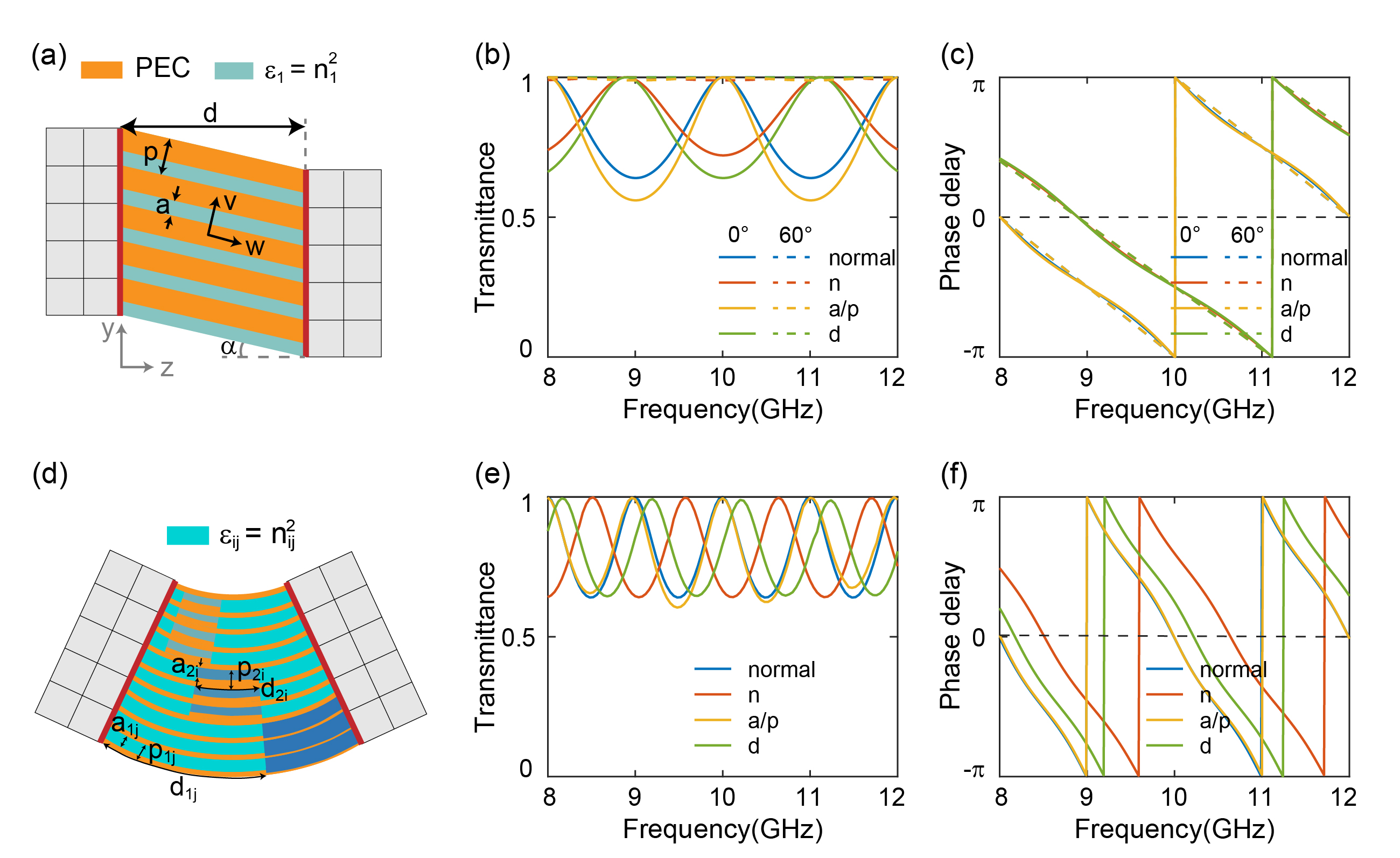
*

**Figure S6.** Sensitivity analysis of device performance to fabrication tolerances. (a) Schematic of a single PEC slot array segment. (b) Simulated transmittance and (c) phase delay for the single segment. These plots compare the normal case (blue line) to cases with an independent -10% perturbation in duty cycle of dielectric ($a/p$, yellow), refractive index of dielectric ($n$, red), and thickness ($d$, green). (d) Schematic of the complete multi-segment cascaded bent waveguide. (e) Simulated transmittance and (f) phase delay for a waveguide with non-uniform optical-path mappings.

To quantify this, we begin with a single PEC slot array shown in Fig. S6a, whose parameters are set to $\alpha=0^{\circ}$, $n=\sqrt{2}$, $d=0.0375 m$, $a/p=\sqrt{2}/2$ (corresponding to a Brewster angle of 60°). Since the perturbation in tilt angle $\delta\alpha$ is typically negligible in practical implementations, we focus on the more common fabrication variations in $n$, $a/p$, and $d$. We applied a -10% perturbation to each parameter independently and simulated the transmittance (Fig. S6b) and phase delay (Fig. S6c) across the X-band. The results reveal three distinct behaviors:

1. **Duty cycle of the dielectric** ($\delta(a/p)$): This primarily affects the impedance matching of the slot array, altering the transmission magnitude. However, it does not shift the FP resonant frequencies, and the transmission phase at resonance remains unperturbed.
2. **Thickness** ($\delta d$): This directly alters the optical path length $nd$, causing a shift in the resonant frequencies. However, it has a negligible effect on the transmission magnitude at non-resonant frequencies.
3. **Refractive index** ($\delta n$): This is the most critical parameter as it simultaneously impacts both the optical path length (shifting resonances) and the impedance matching conditions (reducing transmission magnitude across the entire band)

Notably, the transmission phase profiles for perturbations in $n$ and $d$ are identical, as both result in the same change to the total optical path length. Moreover, at the Brewster incidence, the change in transmittance is minimal for all perturbations, demonstrating the stability of the design at its optimal operating condition.

We extended this analysis to a waveguide with non-uniform optical-path mappings. The structure, whose detailed geometry is provided in Table S1, is shown in Fig. S6d. We introduced a -10% perturbation to the parameters of the dielectric-loaded segments (segments filled with air remained unperturbed). The results in Figs. S6e-f confirm that a perturbation in the refractive index ($\delta n$) causes the largest shift in the resonant frequency and phase error.

Our analysis confirms that the refractive index ($n$) exhibits the highest first-order sensitivity, as a perturbation $\delta n$ simultaneously disrupts both the FP condition and the channel-to-channel impedance matching throughout the cascaded system.

**VII. Designed retroreflector operating at 15 GHz**

In this section, we show how to tune the operating frequency of the retroreflector by adjusting the geometric dimensions of the filling Teflon sheets. Following the same design procedure in Supplementary Data V, we only need to tune the length and thickness of the Teflon sheet in each slot to ensure that the propagation lengths in the filling segments are a multiple of the wavelength and the ones in the air slots are a multiple of the half-wavelength. As an example, the operating frequency is chosen to be 15 GHz, and the revised geometric dimensions of the filling Teflon sheets are shown in Table S3. Note that the geometric dimensions of the copper strips are unchanged.


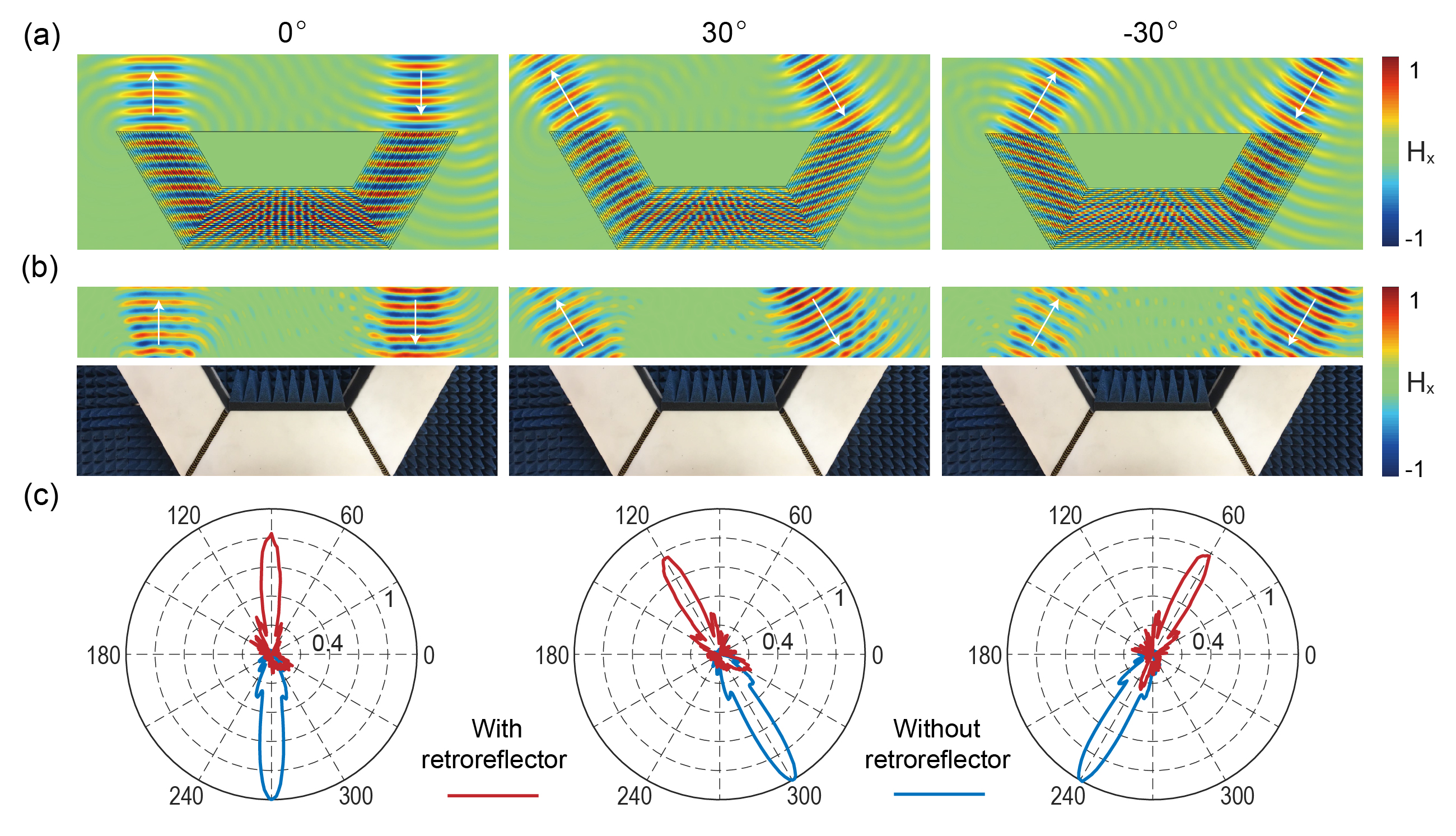


**Figure S7.** Performance of the designed retroreflector operating at 15 GHz. Simulated (a) and measured (b) magnetic field distributions under different incident angles (The incident angle *θ* is 0°, 30°, and -30°, respectively). (c) Measured far-field radiation patterns for three cases with (red lines) and without (blue lines) the retroreflector in front of the transmitting horn antenna.

Fig. S7a shows the simulated magnetic field distributions at 15 GHz when the Gaussian beam is incident on the retroreflector with different incident angles (*θ* = $0^{\circ}$, $30^{\circ}$, and $-30^{\circ}$ for three panels, respectively), and Fig. S7b shows the corresponding measured ones. In the measurements, the field-scanning area is 522 mm × 90 mm with a resolution of 3 mm, and the perimeter of the homemade loop antenna probe is around 10 mm. Fig. S7c shows the measured radiation patterns of the [transmitting](https://cn.bing.com/dict/search?q=transmitting&FORM=BDVSP6&cc=cn) horn [antenna](https://cn.bing.com/dict/search?q=antenna&FORM=BDVSP6&cc=cn) with (red lines) and without (blue lines) the retroreflector in front of it. Normalized by the results without the retroreflector, the calculated retroreflection efficiencies for three cases are 68.9% ($=0^{\circ}$), 57.5% ($=+30^{\circ}$), and 60.5% ($=-30^{\circ}$), respectively, and the reflection angle errors are $0^{\circ}$, $1^{\circ}$, and $0^{\circ}$. These results show that the operating frequency of the designed retroreflector can be easily reconfigured by changing the geometric dimensions of the filling Teflon sheets.

| *Sequence* | *Length of copper strips l_n_ (mm)* | *Filling length f_n_ (mm)* | *The permittivity of the filling dielectric ε_r_n_* | *Filling thickness of Teflon t_n_ (mm)* |
| --- | --- | --- | --- | --- |
| *1* | 560.00 | 35.15 | 1.29 | 0.87 |
| *2* | 551.69 | 66.84 | 1.43 | 1.15 |
| *3* | 543.38 | 18.52 | 1.16 | 0.54 |
| *4* | 535.07 | 50.21 | 1.43 | 1.14 |
| *5* | 526.73 | 121.90 | 1.32 | 0.94 |
| *6* | 518.42 | 33.58 | 1.42 | 1.13 |
| *7* | 510.11 | 105.27 | 1.30 | 0.89 |
| *8* | 501.80 | 16.95 | 1.39 | 1.09 |
| *9* | 493.49 | 88.64 | 1.27 | 0.82 |
| *10* | 485.18 | 120.33 | 1.35 | 1.00 |
| *11* | 476.87 | 72.01 | 1.23 | 0.71 |
| *12* | 468.56 | 103.70 | 1.34 | 0.98 |
| *13* | 460.22 | 55.38 | 1.17 | 0.56 |
| *14* | 451.91 | 87.07 | 1.32 | 0.94 |
| *15* | 443.60 | 38.76 | 1.07 | 0.27 |
| *16* | 435.29 | 70.44 | 1.29 | 0.87 |
| *17* | 426.98 | 102.13 | 1.38 | 1.07 |
| *18* | 418.67 | 53.82 | 1.24 | 0.74 |
| *19* | 410.36 | 85.50 | 1.37 | 1.03 |
| *20* | 402.05 | 37.19 | 1.16 | 0.54 |
| *21* | 393.71 | 68.87 | 1.35 | 1.00 |
| *22* | 385.40 | 100.56 | 1.42 | 1.13 |
| *23* | 377.09 | 52.25 | 1.32 | 0.94 |
| *24* | 368.78 | 83.93 | 1.42 | 1.13 |
| *25* | 360.47 | 35.62 | 1.26 | 0.80 |
| *26* | 352.16 | 67.30 | 1.42 | 1.13 |
| *27* | 343.85 | 18.99 | 1.11 | 0.38 |
| *28* | 335.54 | 50.68 | 1.40 | 1.11 |
| *29* | 327.20 | 82.36 | 1.47 | 1.23 |
| *30* | 318.89 | 34.05 | 1.38 | 1.07 |
| *31* | 310.58 | 65.71 | 1.48 | 1.24 |
| *32* | 302.27 | None | None | None |

**Table S3.** Geometric details of the copper strips and the filling Teflon sheets.

**VIII. Analysis of performance at terahertz and near-infrared frequencies**

In this section, we will discuss the performance of TO devices at Terahertz (THz) frequencies.

The THz band is conventionally defined as 0.1 to 10 THz, where metals exhibit low loss and can be approximated as good electrical conductors. We therefore expect our design to maintain satisfactory performance from 0.5 to 2 THz. To demonstrate, the geometric dimensions of a microwave cloak (Fig. S8a) were scaled down by a factor of 0.01. Fig. S8b presents the simulated transmittance (solid lines) and absorption (dashed lines) spectra of the cloak composed of gold sheets under various incidence angles. Here, the gold is modeled with a Drude model, i.e., *ε*_r_ =1 - *ω*_p_^2^/(*ω*^2^ + *iω*γ). The plasma frequency *ω*_p_ and the damping frequency γ are 1.29×10^16^ rad/s and 7.29×10^13^ rad/s, respectively [2]. For comparison, Fig. S8c shows the transmission when the metal sheet is treated as a perfect electric conductor (PEC). The gold cloak shows marginally lower transmission than the PEC case due to ohmic loss, yet its overall performance remains effective. Meanwhile, the corresponding Brewster angle shows negligible deviation.


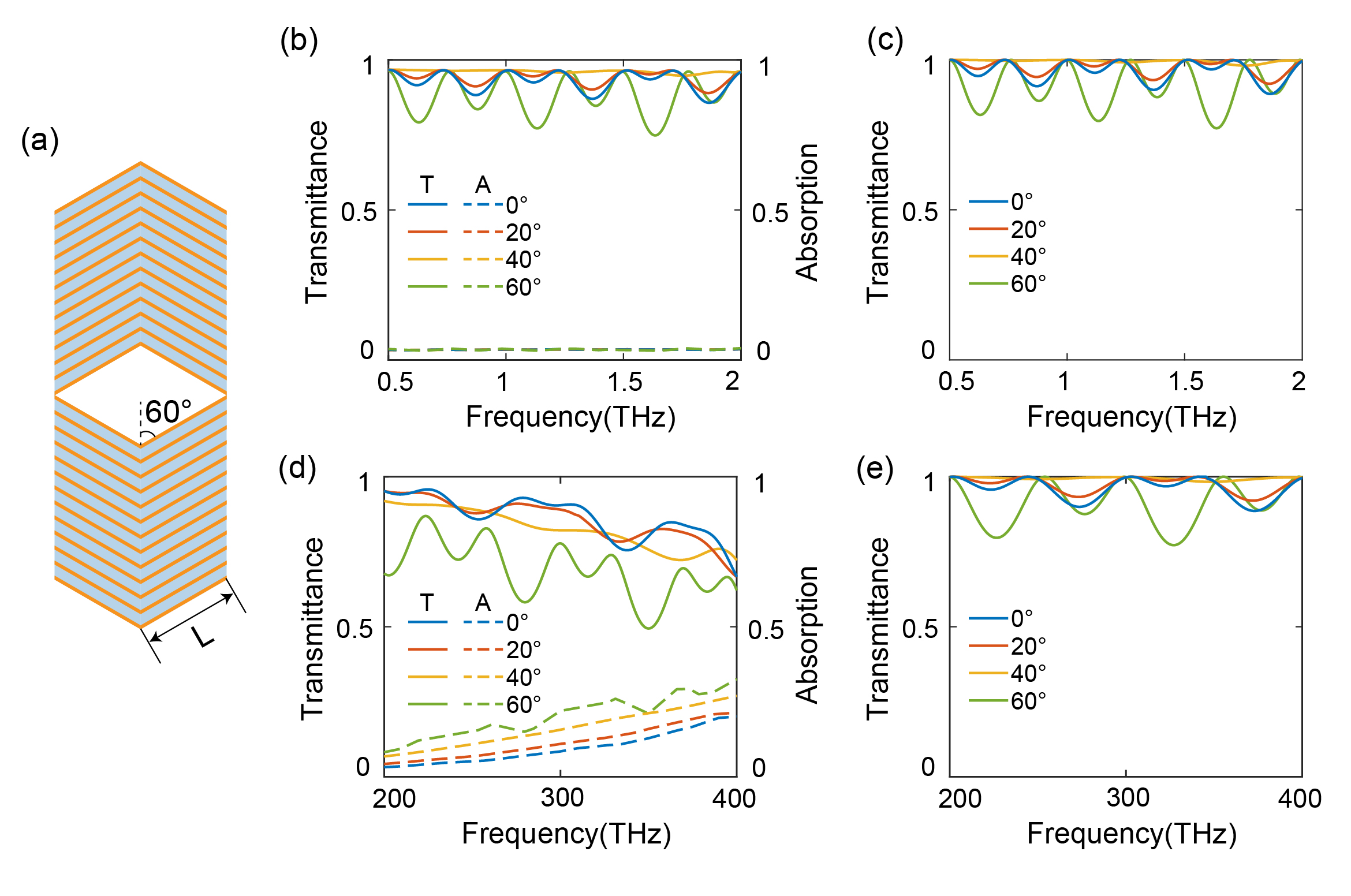


**Figure S8.** Quantitative analysis of material effects on cloak performance at THz. (a) Schematic of the simulated cloak structure. (b) Simulated transmittance (solid lines) and absorption (dashed lines) for gold cloak at 0.5-2 THz. (c) Simulated transmittance for PEC cloak at 0.5-2 THz. (d) Simulated transmittance (solid lines) and absorption (dashed lines) for gold cloak at 200-400 THz. (e) Simulated transmittance for PEC cloak at 200-400 THz.

In contrast, at near-infrared frequencies (200–400 THz), the permittivity of gold changes markedly, leading to substantially higher loss and severe performance degradation. Scaling the original microwave cloak dimensions by 1/30,000, we simulated the cloak again. The transmission spectra for the gold and PEC cloaks are shown in Figs. S8d and S8e, respectively. As anticipated, the gold cloak exhibits strong absorption and an obvious decline in transmission, effectively losing its cloaking function. The Brewster angle is also nearly absent. These results suggest that at such high frequencies, performance cannot be restored to a satisfactory level by structural optimization alone.

**References**

1. Glisson, A. Electromagnetic mixing formulas and applications. *IEEE Antenn Propag Mag* 2000;**42**:72–73.

2. Olmon, R. L. *et al.* Optical dielectric function of gold. *Phys Rev B* 2012;**86**:235147.
